# Nonhuman Primate Induced Pluripotent Stem Cells in Regenerative Medicine

**DOI:** 10.1155/2012/767195

**Published:** 2012-04-19

**Authors:** Yuehong Wu, Anuja Mishra, Zhifang Qiu, Steven Farnsworth, Suzette D. Tardif, Peter J. Hornsby

**Affiliations:** ^1^Department of Physiology and Barshop Institute for Longevity and Aging Studies, University of Texas Health Science Center, 15355 Lambda Drive, San Antonio, TX 78245, USA; ^2^Geriatric Research Education and Clinical Center, South Texas Veterans Healthcare System, San Antonio, TX 78229, USA; ^3^Key Laboratory of Ministry of Education for Protection and Utilization of Special Biological Resources in Western China, and College of Life Science, Ningxia University, Yinchuan, Ningxia 750021, China

## Abstract

Among the various species from which induced pluripotent stem cells have been derived, nonhuman primates (NHPs) have a unique role as preclinical models. Their relatedness to humans and similar physiology, including central nervous system, make them ideal for translational studies. We review here the progress made in deriving and characterizing iPS cell lines from different NHP species. We focus on iPS cell lines from the marmoset, a small NHP in which several human disease states can be modeled. The marmoset can serve as a model for the implementation of patient-specific autologous cell therapy in regenerative medicine.

## 1. Induced Pluripotent Stem Cells in Regenerative Medicine

The aims of regenerative medicine are to restore healthy function to organs damaged by disease or aging. A major issue is the source of cells to be used in regenerative medicine. It is often thought to be desirable to use cells derived from the patient himself/herself, because this is hypothesized to avoid the need to administer drugs to suppress immune rejection of the transplanted cells. The possibility of using patient-specific cells in regenerative medicine was greatly expanded by the discovery of induced pluripotent stem cells (iPS cells) [1, 2]. iPS cells can be derived from any somatic cell, but have the properties of embryonic stem cells. Like embryonic cells, they can be used to generate any cell of the body that may be needed in regenerative medicine. It is widely thought that a form of autologous cell therapy will be possible, in which iPS cells would be derived from the patient's cells, in order to provide a source for cells that could be transplanted back to the patient to restore function to the heart, central nervous system, hematopoietic system, or other organs that are affected by disease or aging. The present experiments concern the development of nonhuman primate models for autologous cell therapy based on iPS cells.

## 2. Autologous versus Allogeneic Cells in Cell-Based Therapies

Any consideration of the implementation of regenerative medicine for human subjects must assess the source of the cells used in the therapy [3, 4]. Following the discovery of iPS cells, it was almost immediately realized that this discovery opened the way to autologous cell therapy. A review in 2007 stated: “If this method can be translated to humans, patient-specific stem cells could be made without the use of donated eggs or embryos” [5]. It is assumed that if the cells are accepted as “self” then they would represent the best possible functional outcome of a transplant: cells that function in their natural environment, without eliciting chronic immune or inflammatory reactions, and without the problems that would result from the use of immunosuppressive drugs. They would, in other words, be the “gold standard” for the best possible results of therapy based on cell transplantation. While allogeneic cells might produce an acceptable result for the patient, autologous cell transplants would provide the standard by which the results of allogeneic cells could be judged.

Shortly after the discovery of iPS cells, the technology was used in a tour-de-force study in which iPS cells were derived from a strain of mice that model human sickle cell anemia. The genetic defect was corrected in the iPS cells and they were transplanted back into mice of the same strain following differentiation to hematopoietic stem cells [6]. The symptoms in the treated mice were substantially ameliorated. This was the first demonstration of the potential power of iPS cell-based therapy. As these cells were derived from, and reintroduced into, mice of the same strain, they are an example of the use of syngeneic cells, rather than truly autologous cells. Subsequently, another study suggested that syngeneic iPS cells and their cell progeny may, in fact, elicit an immune response [7]. This unexpected finding has not yet received a satisfactory explanation. At the date of writing, the question of the immunogenicity of iPS cells and derivatives has only been addressed in mice, and not yet in more translationally relevant species, including primates.

Would therapeutic approaches based on the use of autologous cells be worth the considerable efforts of development and implementation that would be required? The answer at the moment is quite unknown. First, in the absence of suitable translational models, or actual clinical trials of iPS cell-based therapy, the advantages must remain theoretical. We do not know how much better, or not, therapy based on autologous cells would be in comparison to therapy based on allogeneic cells. Possibly, autologous cells will prove to be superior, but perhaps there will be little difference from allogeneic cells. In some therapies, the need for a very rapid treatment would preclude the use of autologous cells. For example, in stroke, due to the need for immediate treatment, “off-the-shelf” cells would be needed and iPS cells are unlikely to be useful. However, understanding whether immune-matched versus mismatched cells would have an advantage in a stroke model would be of great significance.

Second, it is extremely hard to predict how easily-implemented iPS cell-based therapy would eventually become. When iPS cells were first made from skin fibroblasts in 2006-2007, reprogramming was highly inefficient and laborious. Over the last 4 years, there has been astounding progress in terms of better, simpler protocols and increases in efficiency [8–11]. Given that there are no reasons to think that the process should not continue to undergo such improvement in efficiency, it is quite possible that the creation of iPS cells from a patient's cells would become quite routine and inexpensive at some time in the future. Similar dramatic improvements in efficiency and cost have been seen in other biomedical technologies, for example, DNA sequencing.

## 3. Importance of Nonhuman Primate Research in Regenerative Medicine

Before it would be possible to consider applying autologous cell therapy to human patients, the properties of iPS cells must be thoroughly explored in suitable animal models, in order to make sure that autologous cell therapy is both safe and effective. It has been generally recognized that clinically relevant experiments should be performed in a nonhuman primate (NHP) rather than a rodent. NHPs are thought be ideal for such preclinical trials because of their relatedness to humans and their similar physiology, particularly with respect to the central nervous system. Long-term studies of transplanted cell function (>3 years) will be possible in NHPs, but are impossible in rodents.

**Figure 1:**
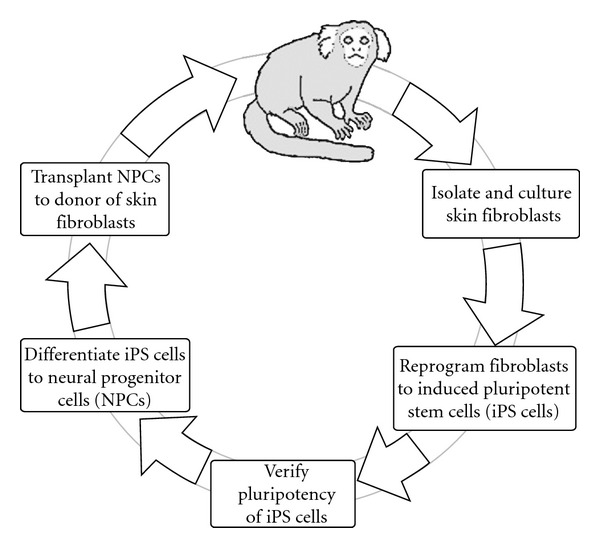
The marmoset as a preclinical model for patient-specific iPS cells in regenerative medicine. This scheme outlines progress to date and future studies of autologous cell transplantation using reprogramming and redifferentiation to a specific cell lineage. A skin biopsy is taken from an individual marmoset, and fibroblasts from the biopsy are grown in culture. Reprogramming factors are expressed in the cells; over a period of several weeks, clones of cells arise that may be iPS cells. Clones are isolated and screened to determine whether they are properly reprogrammed iPS cells; if so, they are expanded and cryopreserved. Neuronal progenitor cells (NPCs) are derived from these iPS cells via protocols described in the text. If the NPCs pass stringent tests of differentiation potential and safety, in the future they may be implanted into the CNS of the same individual from which the cells were originally derived.

Thus there is a clear path from basic to translational studies in iPS cell-based regenerative medicine in NHPs. Of the various NHPs that could be used, the marmoset has several key advantages. The common marmoset (*Callithrix jacchus*) has the advantage of smaller size, more rapid breeding, and defined housing conditions. In contrast to humans, where uncontrolled environment and many comorbidities are confounding factors, marmosets can be housed in a defined environment and have few known comorbidities [12]. A variety of human diseases can potentially be modeled in marmosets [13–15]. A chemical-induced model of Parkinson's disease has also been developed in this species [16] and a stroke model [17] has been developed. Histological and MRI brain atlases are available [18]. The marmoset genome has been completed [19], and the marmoset is the first and so far only primate to have transgenic models that show germline transmission [20]. Although transgenics have also been created in the rhesus macaque, they have not passed the transgene to their offspring [21]. A genetic model of Parkinson's disease by overexpression of *α*-synuclein has been developed in the marmoset [20]. Finally, a spinal cord injury model in the marmoset has been used in tests of transplanted human neural stem cells for potential therapeutic effect [22, 23]. Our long-term goal is illustrated in Figure 1. 

## 4. Progress in NHP iPS Cell Research

Despite the importance of NHPs in regenerative medicine, there has yet been relatively little work on iPS cells derived from NHPs, in comparison to the extent of work on iPS cells derived from mice and humans. The first NHP iPS cells were derived from the rhesus macaque [24]. At the present time (September 2011), iPS cells have been derived from five NHP species (Table 1); three species of macaque (rhesus macaque, pigtailed macaque, and cynomolgus monkey), the common marmoset, and an endangered primate, the drill [24–33]. Common features of all reports on NHP iPS cells are: derivation by mixtures of retroviruses carrying transcription factor cDNAs, principally *POU5F1*, *SOX2*, *KLF4,* and *MYC*; maintenance of pluripotent characteristics over long-term growth in culture; ability to differentiate into cells and tissues of the three germ layers; a lack of malignant properties, despite the ability to form benign teratomas in immunodeficient mice [24–33].

## 5. Marmoset iPS Cells: A Model for Autologous Cell Therapy

The eventual goal of our studies is to derive iPS cells from individual marmosets and implant the cells into the donor animal, following the directed differentiation of the iPS cells to specific cell lineages (Figure 1). Before such studies are possible, extensive in vitro investigations and studies in immunodeficient mice are needed.

We chose to derive marmoset iPS cells from skin fibroblasts because the fibroblast has been the most widely studied cell type for iPS cell generation, and because the use of small skin biopsies as a source of starting material is relevant to future clinical application of iPS cells and their derivatives. In initial experiments, we used fibroblasts derived from newborn marmoset skin [25]. Retroviruses encoding the human cDNAs for Oct4, Sox2, Klf4, and c-Myc [2] were prepared in Plat-A cells and were concentrated by Polybrene flocculation [34]. Following the infection of the cells with concentrated viruses, cultures were maintained in normal fibroblast growth conditions with the addition of valproic acid [35]. After 14–21 days, small colonies of altered morphology were noted in the confluent fibroblast cultures. These colonies comprised small rapidly dividing cells with high nuclear/cytoplasmic ratio and prominent nucleoli. When cultures containing such colonies were fixed and stained for alkaline phosphatase activity, most of the small colonies of altered morphology were found to be positive for alkaline phosphatase, a marker of pluripotency [36]. These colonies expanded rapidly, producing very dense patches of small cells. These cells have the morphological characteristics previously reported for human iPS cells [2].

Starting with a population of 4 × 10^5^ marmoset fibroblasts, we obtained ~100 colonies of cells with iPS cell-like morphology. Colonies were isolated and expanded on feeder layers. Of those colonies that were isolated from the fibroblast cultures, 30 showed sustained growth and were able to be expanded to the point where they could be cryopreserved. Of these, 8 were selected for further study. Karyotypes were investigated by G banding and were found to be normal [25]. Following the initial expansion of marmoset iPS cell clones on feeder layers, we investigated if the cells could be grown under feeder-free conditions. Cells were replated on Matrigel-coated dishes in medium containing 20% fetal bovine serum and 40% MEF-conditioned medium and continued to grow rapidly. Cell populations were expanded under these conditions for further studies.

Marmoset iPS cell clones expressed pluripotency markers at levels that were comparable to that in a human embryonic stem cell line (I6) or exceeded that level [25]. In all 8 marmoset iPS cell clones, NANOG and SOX2 mRNA levels were higher than those in I6 cells, and levels of OCT4 were comparable to that of I6 cells. Levels of OCT4 mRNA were >100-fold higher in iPS cell clones than in the fibroblasts used for reprogramming, and levels of NANOG and SOX2 were >50-fold higher. We assessed the relative levels of vector and total mRNAs for OCT4 and SOX2, two of the factors used for reprogramming. We used primer pairs specific for reprogramming vectors (vector sequence 5′ primer and coding region 3′ primer). Vector OCT4 mRNA was present at 0.01% to 0.1% of that of total OCT4 mRNA, while vector SOX2 mRNA was present at 0.1% to 1% of the total SOX2 mRNA. These findings indicate that the viral genomes are appropriately silenced [38].

In order to assess the potential of marmoset iPS cell clones to differentiate to cells of all three germ layers, cells were transplanted into immunodeficient mice (subcutaneous injection in 50% Matrigel: [39, 40]). Teratomas from marmoset iPS cells contained a variety of tissue structures representing derivatives of all three germ layers. Because it has been reported that teratomas derived from incompletely reprogrammed cells formed tissues of ectodermal and mesodermal origin but not of endodermal origin [38] we performed histological studies of the development of mature structures of endodermal origin; we observed endodermal tissues, including simple columnar and pseudostratified epithelia, epithelia with goblet cells, and exocrine glandular structures [25]. Immunohistochemical studies were also performed; ectodermal tissue (developing neural tissue) was demonstrated by presence of *β*III tubulin; mesodermal tissue by smooth muscle actin; endodermal tissue by *α*-fetoprotein.

**Figure 2:**
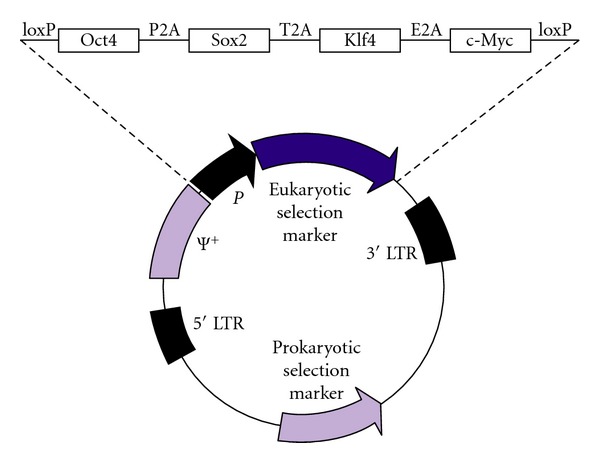
Retroviral reprogramming vector designed to deliver four reprogramming factors (Oct4, Sox2, Klf4, and c-Myc; OSKM) in a single virus using “self-cleaving” peptides, which support efficient polycistronic expression from a single promoter [8]. In this version, expression is driven by the 5′ LTR. Additionally, loxP sites are present just before and just after the OSKM coding region, enabling excision of the vector from the genome of the reprogrammed cells. This vector was constructed by replacing the internal promoter (P) and eukaryotic selection marker of retroviral vector pLXSN by the OSKM sequence from FUW-OSKM [8].

Subsequently, we investigated the potential of a polycistronic vector for reprogramming (Figure 2). This retroviral vector has the features that (a) because expression of the reprogramming factors is driven by the 5′ LTR, expression is silenced during reprogramming, if cells have been properly reprogrammed [38]; (b) all factors are in one vector, thus avoiding the need for very high efficiency infection; (c) as a retroviral vector, only dividing cells are infected (this does not detract from the value of this type of vector, as iPS cells must arise from cells capable of cell division); (d) loxP sites enable future excision of the coding region when required. Marmoset iPS cells derived using this polycistronic retroviral vector exhibited the same characteristics of iPS cell clones derived by coinfection of the four factors. Therefore, cells derived by a 1 : 1 : 1 : 1 expression of the four reprogramming factors have properties that are basically the same as those derived by coinfection, in which the ratio of expression of the four factors is not necessarily equal and almost certainly varies from clone to clone.

Despite the advantages of such retroviral vectors, it is likely that the use of integrating forms of viral vectors for reprogramming will be made obsolete by nonviral reprogramming methods using modified mRNA or modified proteins [9]. These methods avoid any genetic modification of the target cells during the reprogramming process.

**Figure 3:**
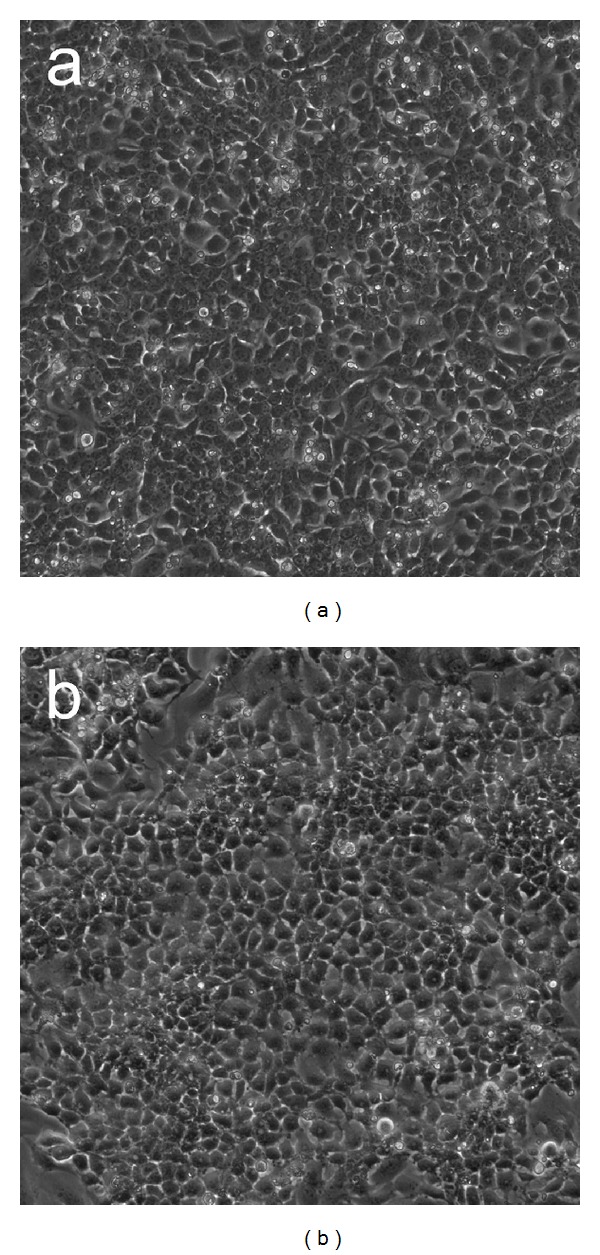
Marmoset iPS cells growing in feeder-free culture. (a) An iPS cell line derived by coinfection with four retroviruses (B8 cell line [25]). Cells are growing in defined xeno-free medium (Pluriton, Stemgent). (b) An iPS cell line derived by infection with a single retrovirus, encoding the OSKM reprogramming factors, illustrated in Figure 2.

Successful long-term expansion of marmoset iPS cells is critical for any extensive studies of the properties of the cells. Although we determined feeder-free conditions for growth of the cells, these conditions require fetal bovine serum and medium conditioned by a suitable cell type, such as mouse embryo fibroblasts. More recently, we have established that marmoset iPS cells can grow continuously and over long periods in defined medium without the addition of serum or of medium conditioned by another cell type. Several types of defined media support long-term marmoset iPS cell growth without loss of expression of pluripotency genes such as NANOG and OCT4/POU5F1. Both clones derived by coinfection and clones derived by infection with a polycistronic vector may be grown in defined medium (Figure 3).

In summary, by the criteria of morphology, growth requirements, expression of pluripotency factors, retroviral silencing, and the ability to generate teratomas with tissues of all three germ layers, we conclude that these lines of cells represent *bona fide *induced pluripotent stem cells.

## 6. Differentiation of Marmoset iPS Cells to Neural Progenitor Cells

**Figure 4:**
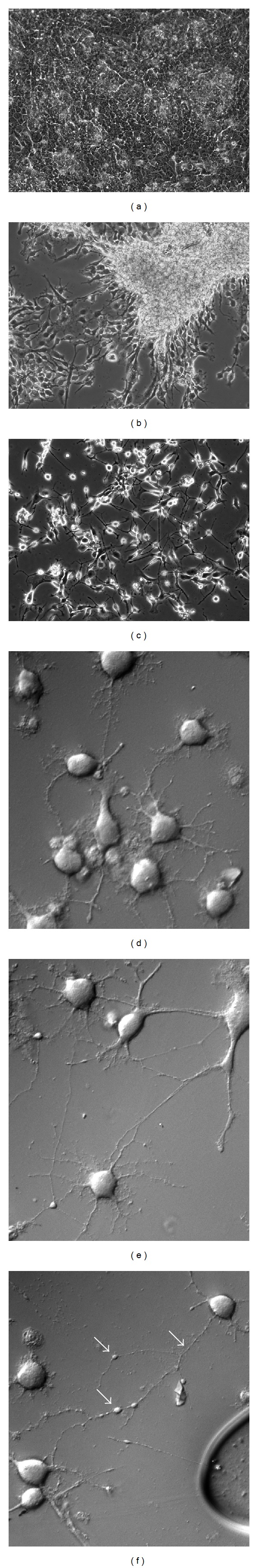
Derivation of neural progenitor cells (NPCs) from marmoset iPS cells and differentiation of NPCs to mature neurons. The series (a)–(c) shows the transition from undifferentiated iPS cells (a), to a line of NPCs (b), to mature neurons (c) (100x phase-contrast images). NPCs placed on a polylysine/laminin-coated glass surface stop dividing and form extensive axons and dendrites. Details of this further maturation are shown in series (d)–(f) (400x differential interference contrast images). Note particularly the varicosities of different sizes indicated by arrows in (f). These are sites of accumulation of cellular organelles and are precursors to the formation of synapses [37]. Their presence indicates the degree of maturity of these neurons.

In subsequent work, we investigated the potential of marmoset iPS cell lines to differentiate in vitro to cells of the neural lineage. Differentiation of iPS cells to neural progenitor cells (NPCs) has been extensively employed as a test of proper pluripotency; for example, this form of directed differentiation was used in a recent set of tests on a panel of well characterized human iPS cells [10, 11]. Protocols for NPC generation are of three general types: stromal cell-derived inducing activity (SDIA), a relatively poorly characterized mix of factors secreted by certain mesenchymal cells, such as the PA6 cell line [2, 41, 42]; embryoid body (EB) formation, followed by plating of the EBs on suitable surfaces in the presence of Neurobasal medium [43, 44]; and induction using small molecules, such as chemical inhibition of BMP/activin/nodal signaling via SMADs [45]. We have used each of these methods in marmoset iPS cells, and all of them produce NPC lines (Figure 4).

## 7. Summary

In summary, iPS cells from NHPs have a unique importance in preclinical research leading to the implementation of regenerative medicine in human patients. We have derived and characterized iPS cells from the marmoset, a small NHP that can serve as a suitable model for autologous cell therapy involving iPS cells. Future studies will test the principles of autologous cell therapy in individual marmosets.

## Figures and Tables

**Table 1:** Publications on nonhuman primate iPS cells.

Species	Title of publication	cDNAs used for reprogramming	Origin of cDNAs
Rhesus macaque (*Macaca mulatta*)	Generation of induced pluripotent stem cells from adult rhesus monkey fibroblasts [24]	*POU5F1, SOX2 KLF4* and *MYC *	Rhesus
Common marmoset (*Callithrix jacchus*)	Generation of induced pluripotent stem cells from newborn marmoset skin fibroblasts [25]	*POU5F1, SOX2 KLF4* and *MYC *	Human
Common marmoset (*Callithrix jacchus*)	Generating induced pluripotent stem cells from common marmoset (*Callithrix jacchus*) fetal liver cells using defined factors, including Lin28 [26]	*POU5F1, SOX2, KLF4, MYC, NANOG* and *LIN28 *	Human
Rhesus macaque (*Macaca mulatta*)	Reprogramming Huntington monkey skin cells into pluripotent stem cells [27]	*POU5F1, SOX2* and *KLF4 *	Rhesus
Pigtailed macaque (*Macaca nemestrina*)	Efficient generation of nonhuman primate induced pluripotent stem cells [28]	*POU5F1, SOX2 KLF4* and *MYC *	Human
Cynomolgus monkey (*Macaca fascicularis*)	Development of histocompatible primate induced pluripotent stem cells for neural transplantation [29]	*POU5F1, SOX2 KLF4* and *MYC *	Human
Rhesus macaque (*Macaca mulatta*)	Generation of pancreatic insulin-producing cells from rhesus monkey induced pluripotent stem cells [30]	*POU5F1, SOX2 KLF4* and *MYC *	Rhesus
Pigtailed macaque (*Macaca nemestrina*)	Safeguarding nonhuman primate iPS cells with suicide genes [31]	*POU5F1, SOX2 KLF4* and *MYC *	Human
Drill (*Mandrillus leucophaeus*)	Induced pluripotent stem cells from highly endangered species [32]	*POU5F1, SOX2 KLF4* and *MYC *	Human
Cynomolgus monkey (*Macaca fascicularis*)	Induction of retinal pigment epithelial cells from monkey iPS cells [33]	*POU5F1, SOX2 KLF4* and *MYC *	Human

The table lists the publications (in order of publication, up to September 2011) that have reported the derivation and characterization of nonhuman primate iPS cells. All used mixtures of retroviruses, carrying the indicated cDNAs.
